# The Assessment of Disease Activity in Rheumatic Diseases 

**DOI:** 10.1155/2013/275691

**Published:** 2013-11-07

**Authors:** Thurayya Arayssi, Zahi Touma, Mandana Nikpour, Lilian Ghandour

**Affiliations:** ^1^Weill Cornell Medical College in Qatar, Doha 24144, Qatar; ^2^Centre for Prognosis Studies in the Rheumatic Diseases, University of Toronto, Toronto, ON, Canada M5T 2S8; ^3^The University of Melbourne, St. Vincent's Hospital, 41 Victoria Parade Fitzroy, Melbourne, VIC 3065, Australia; ^4^Faculty of Health Sciences, American University of Beirut, Beirut 1107 2020, Lebanon

Disease activity in the rheumatic diseases can be defined as a reversible state, manifested by clinical, laboratory, or radiographical features. Disease activity occurs principally as a result of immunologic and inflammatory processes involving specific organs at a specific point in time. The multifaceted nature of clinical presentations in adults as well as in pediatric rheumatic diseases makes the assessment of disease activity challenging. Nevertheless, this assessment is fundamental in patient care and based on the use of valid, reliable, and interpretable instruments. The use of disease activity instruments enables clinicians, patients, and researchers to quantify and evaluate disease activity in a standardized way. The application of these instruments in a clinical care and research setting presents several challenges: namely, the administrative burden of the instrument, including the preparedness and skillfulness of the assessor, the mode of administration, the time required to complete the instrument, and the complexity of scoring. All of these factors need to be taken into consideration when choosing an instrument applicable in a particular setting.

In the last decade, we have witnessed the emergence of a plethora of instruments developed to measure disease activity in the rheumatic diseases. This special issue contains five papers: four related to the assessment of activity in adult rheumatic diseases and one paper with a focus on pediatric rheumatic disease. 

Wong et al. “*Measuring disease activity in psoriatic arthritis*” provide a detailed review of the assessment of disease activity in psoriatic arthritis. Their paper highlights the currently available tools for the assessment of the various rheumatologic and dermatologic aspects of the disease and discusses the composite indices that have been developed, or are still under development.

Dowsey and Choong “*The utility of outcome measures in total knee replacement surgery*” discuss the utility of outcome measures for total knee replacement surgery, most commonly performed to treat osteoarthritis. While the majority of patients have a substantial improvement in symptoms following this procedure, a proportion of patients report ongoing pain and poor function. The authors remind us that accurate and reproducible measurement of residual pain and functional impairment in patients undergoing arthroplasty forms an important basis for optimizing outcomes of this procedure. The paper by Dowsey et al. challenges our perception of disease “activity” in the rheumatic diseases and allows us to consider the overlap with the broader concept of “severity,” particularly in osteoarthritis, which does not traditionally follow a relapsing-remitting course. 

In an original research article, Ohrndorf et al. “*Detailed joint region analysis of the 7-joint ultrasound score: evaluation of an arthritis patient cohort over one year*” use ultrasound technique, specifically the 7-joint ultrasound (US7) score, to evaluate disease activity and the presence of erosions over one year in a cohort of patients with predominantly rheumatoid and psoriatic arthritis. They demonstrate a high synovitis score at baseline in the majority of patients, which diminishes over one year of treatment, corresponding with a decline in the DAS28 score. The responsiveness over time of ultrasound-based assessment of disease activity in inflammatory arthritis, compared with clinical-laboratory based assessment, and the prognostic significance of mild persistent synovitis detectable on ultrasound are yet to be conclusively demonstrated and quantified. However, the study by Ohrndorf et al. highlights the potential usefulness of imaging modalities in assessment of activity in the rheumatic diseases. 

Quimby et al. “*Comparison of the systemic lupus erythematosus activity questionnaire and the systemic lupus erythematosus disease activity index in a black barbadian population*” evaluate the use of the Systemic Lupus Activity Questionnaire (SLAQ), vis-à-vis the internationally accepted and validated Systemic Lupus Erythematosus Disease Activity Index (SLEDAI), and the physician global assessment (PGA). They conclude that SLAQ results in over-reporting of symptoms and is an inadequate disease monitoring tool. They underscore the use of laboratory measurements and highlight the need for a modified version that would be valid, feasible, and available to countries most burdened by SLE, yet least equipped to diagnose and manage the disease. 

Finally, Luca's and Feldman's “*Disease activity measures in paediatric rheumatic diseases*” provide a comprehensive review of assessment of disease activity in the pediatric population. Their paper explains the theoretical framework required for the development of disease activity measures citing the challenges encountered in this particular patient population. In addition, they summarize the most common disease activity measures used for Juvenile Idiopathic Arthritis (JIA), Juvenile Systemic Lupus Erythematosus (JSLE), and Juvenile Dermatomyositis (JDM) reminding us that more research is needed to determine the most appropriate measures to be used in clinical practice and the research environment.

The assessment of disease activity in rheumatic diseases is very challenging but essential. Significant achievements have been made in improving the measurement properties of the instruments. Further research is needed to identify the measurement tools that are of the highest quality and can be conveniently used in clinics and in research studies.

